# Network analysis-based strategy to investigate the protective effect of cepharanthine on rat acute respiratory distress syndrome

**DOI:** 10.3389/fphar.2022.1054339

**Published:** 2022-10-26

**Authors:** Chen Chen, Ning Wang, Bingjie Wang, Qiaoyun Zhang, Yuexia Hu, Gao Cheng, Shaoyi Tao, Jian Huang, Chunhui Wang, Ye Zhang

**Affiliations:** ^1^ Department of Anesthesiology, The Second Affiliated Hospital of Anhui Medical University, Hefei, China; ^2^ Key Laboratory of Anesthesiology and Perioperative Medicine of Anhui Higher Education Institutes, Anhui Medical University, Hefei, China; ^3^ Anhui Public Health Clinical Center, Hefei, China; ^4^ Department of Anesthesiology, The First Affiliated Hospital of Anhui Medical University, Hefei, China; ^5^ Department of Thoracic Surgery, First Affiliated Hospital of Anhui Medical University, Hefei, Anhui, China

**Keywords:** cepharanthine, ARDS, inflammation, traditional Chinese medicine, network analysis

## Abstract

Combined with Network Analysis (NA) and *in vivo* experimental methods, we explored and verified the mechanism of Cepharanthine (CEP) involved in the treatment of acute respiratory distress syndrome (ARDS). Potential targets of CEP were searched using the SwissTargetPrediction database. The pathogenic genes related to ARDS were obtained using the DisGeNET database. A protein-protein interaction network of common target genes of disease-compound was subsequently built and visualised. Functional enrichment analysis was performed through the Enrichr database. Finally, for *in vivo* experimental verification, we established an oleic acid-induced ARDS rat model, mainly through histological evaluation and the ELISA method to evaluate both the protective effect of CEP on ARDS and its effect on inflammation. A total of 100 genes were found to be CEP targeted genes, while 153 genes were found to be associated with ARDS. The PPI network was used to illustrate the link and purpose of the genes associated with CEP and ARDS, which contained 238 nodes and 2,333 links. GO and KEGG analyses indicated that inflammatory response and its related signalling pathways were closely associated with CEP-mediated ARDS treatment. Thus, a key CEP–gene–pathway-ARDS network was constructed through network analysis, including 152 nodes (5 targets and 6 pathways) and 744 links. The results of *in vivo* experiments showed that CEP could alleviate histopathological changes and pulmonary edema related to ARDS, in addition to reducing neutrophil infiltration and secretion of inflammatory cytokines, whilst increasing serum contents of ResolvinD1 and ResolvinE1. Thus, these effects enhance the anti-inflammatory responses. Thus, our results show that CEP can treat oleic acid-induced ARDS in rats *via* ResolvinE1 and ResolvinD1 signalling pathways that promote inflammation resolution, providing a new avenue to explore for the clinical treatment of ARDS.

## 1 Introduction

Acute respiratory distress syndrome (ARDS) is a rapidly progressing disease, which can be divided into two categories according to its underlying diseases: direct ARDS is a result of lung pathology, whilst indirect ARDS is caused by systemic inflammation (Monahan, 2013; Dickson et al., 2016). ARDS is a refractory disease associated with a high mortality (van Gemert et al., 2021). In the ICU, ARDS was reported to be the cause of 10.4% of admissions. Up to the 2000s, the mortality rate for ARDS was reported to be as high as 40%–70%. (Mane and Isaac, 2021). Although several ARDS treatments have been developed, none show the efficacy needed to reduce mortality or prolong the lives of ARDS patients.

Traditional Chinese medicine (TCM) has been widely used to treat lung diseases (Tsai et al., 2013). Relevant studies have shown that TCM has obvious effects in alleviating ARDS pulmonary inflammation, reducing mortality and improving prognosis (Yeh et al., 2021; Ting et al., 2022). Cepharanthine (CEP), a monomer component of TCM, is a natural alkaloid extracted from *Step* *hania cepharantha Hayata*, which has anti-inflammation, immunomodulation, antioxidation, anti-parasitic and anti-virus effects (Murakami et al., 2000; Furusawa and Wu, 2007; Kudo et al., 2011). Rat pharmacokinetics demonstrate that after a single dose of intravenous administration of 1 mg/kg, CEP achieved a 153.17 ± 16.18 ng/ml maximum plasma concentration and the *t*_1/2_ was 6.76 ± 1.21 h. Bioavailability of CEP in rats after administration oral bioavailability of CEP was 5.65% ± 0.35%, which showed that oral bioavailability was low (Deng et al., 2017). As early as 2020, Tong et al. (Fan et al., 2020) has reported the research results of anti-Coronavirus Disease 2019 drugs, and found that CEP is a potential drug for the treatment of severe acute respiratory syndrome coronavirus 2 (SARS-CoV-2) infection. Recently, the scientific research team in China has successfully obtained the national invention patent authorization of CEP (Jiang et al., 2022). The patent specification shows that CEP 10 μmol/L inhibits SARS-CoV-2 replication by 15,393 times, showing a strong ability to inhibit viruses (Fan et al., 2020). Although CEP has the aforementioned beneficial characteristics, its role in the treatment of ARDS has not been reported. Network Analysis (NA) is a cross-discipline, that is, based on system biology, combining polypharmacology, molecular network data, bioinformatics and computer simulation (Gao et al., 2020; Sakle et al., 2020). It uses the database information of drugs, compounds, genes, and diseases to construct the interaction network of drug targets, disease targets and signal pathways, so as to reveal the complex mechanism of multi-components and multi-target characteristics of TCM (Saberian et al., 2019; Zhang et al., 2021). It provides the basis for the transformation of TCM from empirical medicine to evidence-based medicine, and provides some guidance for the development and application of new clinical drugs.

Based on the above research background, this study used NP to predict the potential target and pathway of CEP in the treatment of ARDS. We carried out *in vivo* experiments to verify the mechanism of CEP in the treatment of ARDS, which provided a research foundation for its clinical application.

## 2 Materials and methods

### 2.1 Absorption, distribution, metabolism and excretion screening

Common ADME characteristics include lipophilicity, water solubility, pharmacokinetics and drug-likeness (Nainwal et al., 2020; Nabi et al., 2022). We looked into the properties of CEP’s ADME using the SwissADME database. Since 2017, SwissADME has been a Web application that provides free access to a collection of quick, yet reliable, predictive models to assess physicochemical properties, pharmacokinetics, drug-likeness, and synthetic accessibility, including in-house effective techniques like iLOGP (a physics-based model for lipophilicity) (Daina et al., 2017). The 2D structure of CEP is obtained by using chemdraw software (Ultra 8.0).

### 2.2 Acquisition target genes of cepharanthine

Utilising the PubChem (https://pubchem.ncbi.nlm.nih.gov) website, the structural formula of CEP was obtained in the Canonical SMILES format, and used as input for SwissTargetPrediction free webserver to predict potential molecular targets of CEP.

### 2.3 Disease-related target genes

The pathogenic genes related to ARDS were obtained by using the DisGeNET database (Piñero et al., 2021), which is one of the largest publicly accessible datasets of genes and variations linked to human disorders. Data from expert-curated sources, GWAS catalogues, animal models, and scientific literature are all combined *via* DisGeNET. DisGeNET data are uniformly labeled using community-driven ontologies and controlled vocabularies. A number of unique measures are also offered to help with prioritising genotype-phenotype connections.

### 2.4 Common target genes of disease-compound

By creating venn diagrams, the targets of ARDS were intersected with the targets of active compound. Finally, the common targets of ARDS and the potential targets of CEP (in the treatment of ARDS) were obtained.

### 2.5 Discovery of protein-protein interactions network

To identify the network PPI, we utilised STRING11.0 (Szklarczyk et al., 2017) (https:/string-db.org/), an online tool for investigating protein interactions. The “Homo sapiens” sample type was chosen, and all gene symbols for ARDS and CEP were entered. For the PPI network development, a minimum interaction score requirement of 0.7 was used, and unconnected nodes were buried in the network for future presentation. The initial information on protein interactions was downloaded. Cytoscape v3.9.1 (Shannon et al., 2003) (https://www.cytoscape.org/), a tool used to visually analyse networks of protein interaction, portrayed the nodes and links in networks of protein interaction; this was used to visualise the PPI network.

### 2.6 Functional analysis

Gene Ontology (GO) enrichment analysis of biological processes (BP), cellular composition (CC), and molecular function (MF) of the common target genes of disease-compounds identified above were carried out using the Enrichr database (Kuleshov et al., 2016). Also examined were the common target genes pertaining to the Kyoto Encyclopedia of Genes and Genomes (KEGG) pathways and Jensen tissue. The analysis cutoff threshold, *p* < 0.05, was established.

### 2.7 Construction of key CEP–gene–pathway-acute respiratory distress syndrome network

Based on the aforementioned analysis, a CEP–gene–pathway-ARDS network was constructed using Cytoscape v3.9.1.

## 3 *In vivo* experimental verification

### 3.1 Experimental animals

Fifteen Sprague-Dawley rats (320 ± 30 g) were used as experimental animals in this study. They were randomly divided into three groups: Control (*n* = 5), ARDS (*n* = 5) and ARDS + CEP (*n* = 5). During the experiment, animals were placed in enriched cages with access to water and food. The cage was a temperature and hygrometry-controlled vicinity. The study protocol was approved by the Laboratory Animal Ethics Committee of Anhui Medical University (No. LLSC20190476) and performed according to the ARRIVE guidelines (https://www.nc3rs.org.uk/arrive-guidelines) for animal experiments.

### 3.2 Experimental protocol

CEP (purity 99.82%, Cat# HY-N6972, Selleck, Huston, TX, United States) was dissolved in sterile saline to a final concentration of 1.07 × 10^4^ μmol/mol prior to the experiment. The rats were anaesthetised with pentobarbital (30 mg/kg intraperitoneally). After local disinfection of the right groin, 0.5% lidocaine anaesthesia was injected, penetrating layer-by-layer, in order to expose and separate the right femoral artery and vein. Next, a rat ARDS model was established according to the previous study (Li et al., 2021; Huang et al., 2022a; Huang et al., 2022b). Highly pure (99.9%) oleic acid (100 mg/kg) (Sigma, St. Louis, MO, United States) was slowly injected into the rat body through the femoral vein with a microsyringe. CEP (10 mg/kg) was injected intraperitoneally 1 h after oleic acid injection in the ARDS + CEP group, and the dose was determined according to previous reports (Murakami et al., 2000; Kudo et al., 2011; Chang et al., 2016). The Control and ARDS groups were injected with the same amount of sterile saline. 24 h after the intraperitoneal injection, rats were sacrificed by CO_2_ narcosis and cervical dislocation. Subsequently, broncho-alveolar lavage fluid (BALF), in addition to serum and lung tissues, were collected for use in subsequent experiments.

### 3.3 BALF protein concentration and the Wet/Dry weight ratio

After the left main bronchus was clamped, BALF of the right lung was performed with 2 ml of pre-cooled sterile saline through a tracheal cannula. According to the manufacturer’s instructions, we measured the BALF protein concentration using a bicinchoninic acid protein assay kit (Solaibao, Beijing, China). In the measurement of the Wet/Dry ratio, the left lower lobe of the lung was taken and weighed wet. The lung dry weight was recorded after being placed in an oven for 48 h. The Wet/Dry weight ratio of lung was subsequently calculated.

### 3.4 Histological evaluation

A paraffin embedding procedure was performed on the left superior lobes of the lung that were fixed in 10% buffered formalin for 48 h. Hematoxylin-eosin (H&E) staining and Immunohistochemical (IHC) staining was performed on 5 μm paraffin-embedded sections. For H&E staining, paraffin-embedded tissues were de-waxed, rehydrated, HE stained, and dehydrated. The lung sections were scored by a pathologist in a blinded fashion. The degree of lung injury was scored based on the following variables: hemorrhage, lung edema, inflammatory cell infiltration, hyaline membrane, and atelectasis. For IHC analysis, sections were incubated with Myeloperoxidase (MPO) (1:200, Cat# ab208670, Abcam, United States) antibodies at 4°C overnight followed by the secondary antibody. Diaminobenzidine substrate kits (Vector Laboratories, Burlingame, CA) were used to reveal the IHC reaction. For cell count, 10 high-power fields at a magnification of ×400, were randomly selected, and ∼200 cells were counted in each field. All the sections were observed under an optical microscope.

### 3.5 Enzyme-linked immunosorbent assay

After the experiment, rat blood was collected from the inferior vena cava, then centrifuged at 3500 rpm for 15 min at 4°C. To assess the degree of inflammation, level of inflammatory cytokines such as TNF-α, IL-1β, IL-6, IL-8, ResolvinD1 (ResD1) and ResolvinE1 (ResE1) in the serum, lung and BALF were measured using ELISA assay kit (Bioexcellence, Beijing, China) according to the manufacturer’s instructions.

### 3.6 Statistical analysis

We used the SPSS 22.0 statistical software (SPSS Inc., Chicago, IL, United States) to carry out statistical analysis and we created graphs using GraphPad Prism 7.0 (GraphPad, United States). Measurement data were expressed as mean ± standard deviation (SD). The normality of distribution was studied using the Shapiro-Wilk test. Independent sample *t*-test and/or one-way ANOVA were used to compare data between the two groups. *p* values <0.05 were considered statistically significant.

## 4 Results

### 4.1 Evaluation of acute respiratory distress syndrome parameters of CEP

**FIGURE 1:**
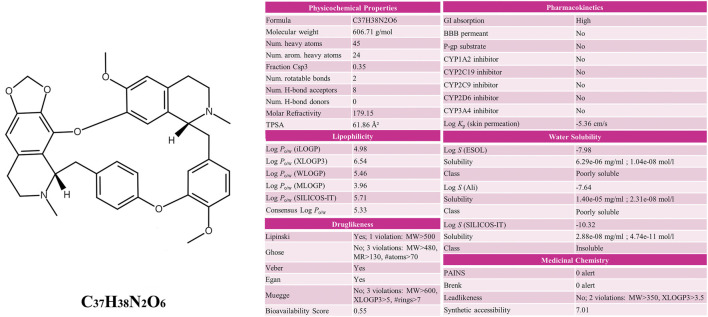
Chemical structure and ADME parameters of CEP.

CEP is a bisbenzylisoquinoline alkaloid from *Stephania cepharantha Hayata,* whose chemical formula is C_37_H_38_N_2_O_6_. Through the SwissADME database, the associated ADME characteristics of CEP were examined. SwissADME screening criteria: a. Gastrointestinal absorption (GI absorption) is “high”; b. 2 out of 5 gastric drug properties (Lipinski, Ghose, Veber, Egan, Muegge) results are “yes”; c. Lipophilicity is less than 5.5; d. Water solubility is less than −6. The findings revealed that the CEP’s lipophilicity value was 5.36 and its water solubility value was −7.98. GI absorption was high, and the bioavailability score was 0.55. These values demonstrate the compound’s enormous potential to be develop as a therapeutic molecule (Figure 1).

### 4.2 Identification of common target genes of disease-compound

**FIGURE 2:**
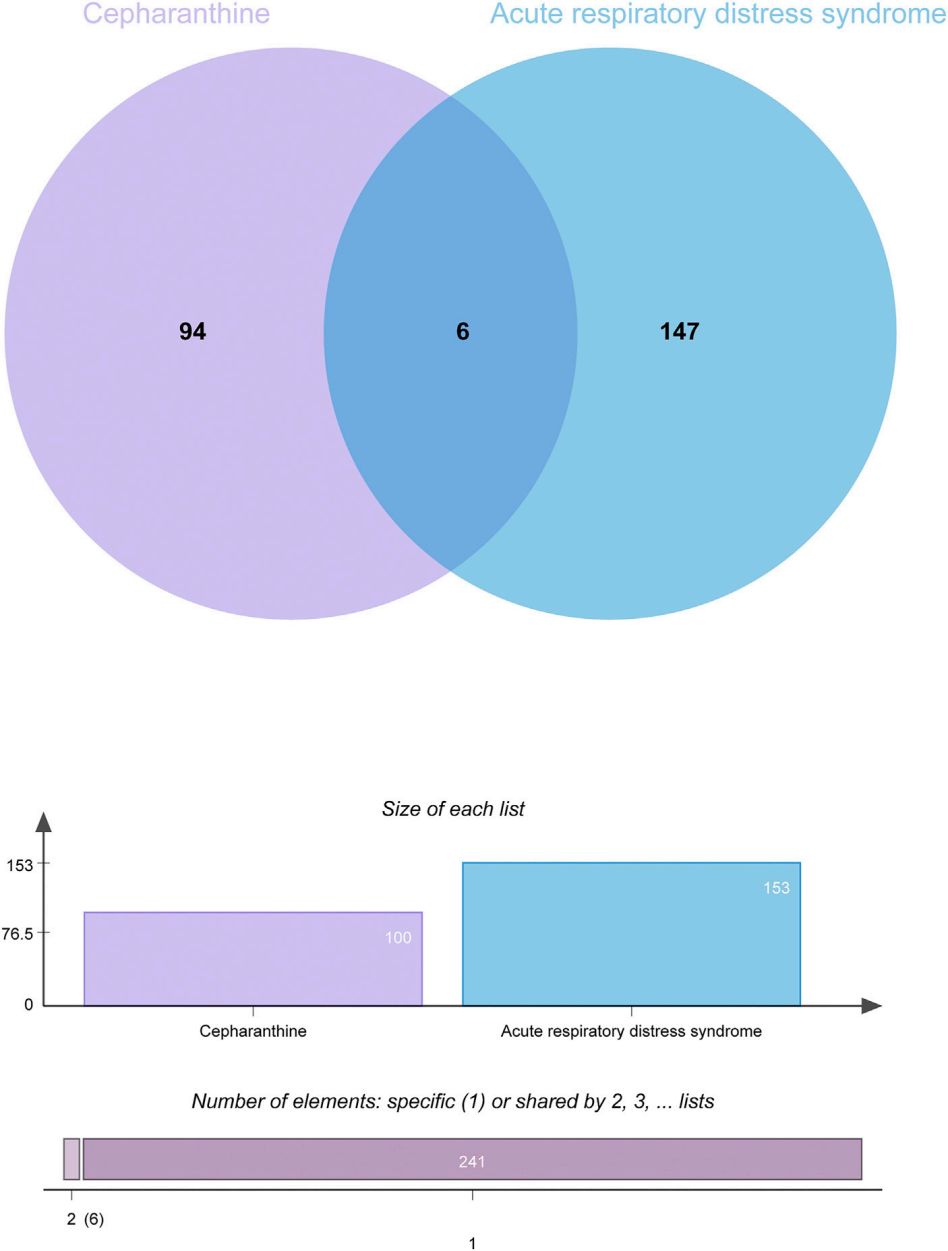
Venn diagram of the interactions of CEP targeted genes and genes associated with ARDS.

A total of 100 genes were found to be CEP-targeted genes, while 153 genes were found to be associated with ARDS. A total of 6 genes were identified by the intersection between CEP’s target genes and ARDS-related genes. In Figure 2, the target genes of disease-compounds were shown.

### 4.3 Construction of the PPI network

**FIGURE 3:**
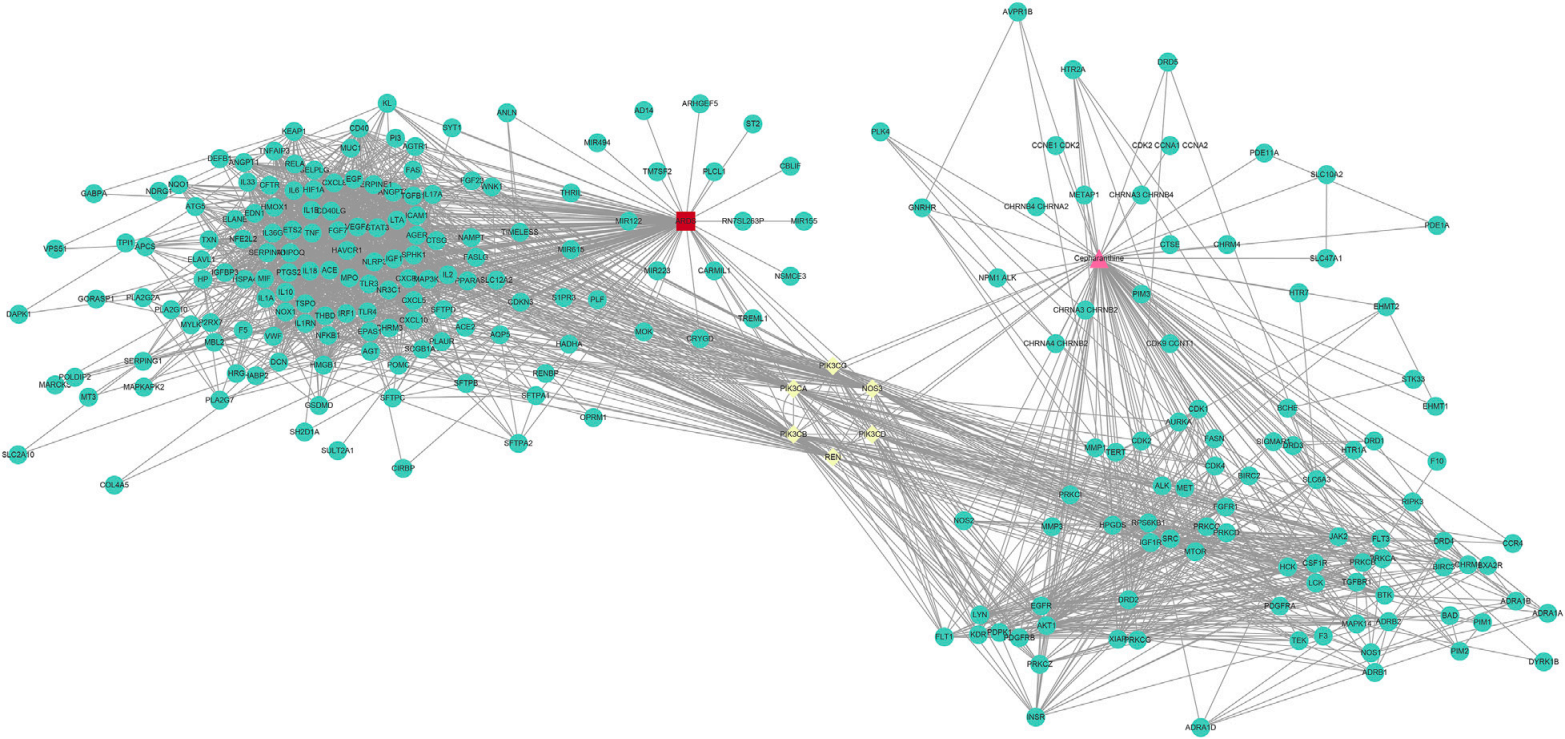
PPI network of CEP’s targeted genes and genes associated with ARDS.

To collect PPI information, the discovered genes were submitted to the STRING database. In order to create the PPI network, we utilised Cytoscape (version 3.9.1). The PPI network (developed after the elimination of isolated nodes) was used to illustrate the link and purpose of the genes associated with CEP and ARDS. There were 238 nodes and 2333 links in the PPI network (Figure 3).

### 4.4 GO enrichment analysis and tissue analysis of common target genes of disease-compounds

**FIGURE 4:**
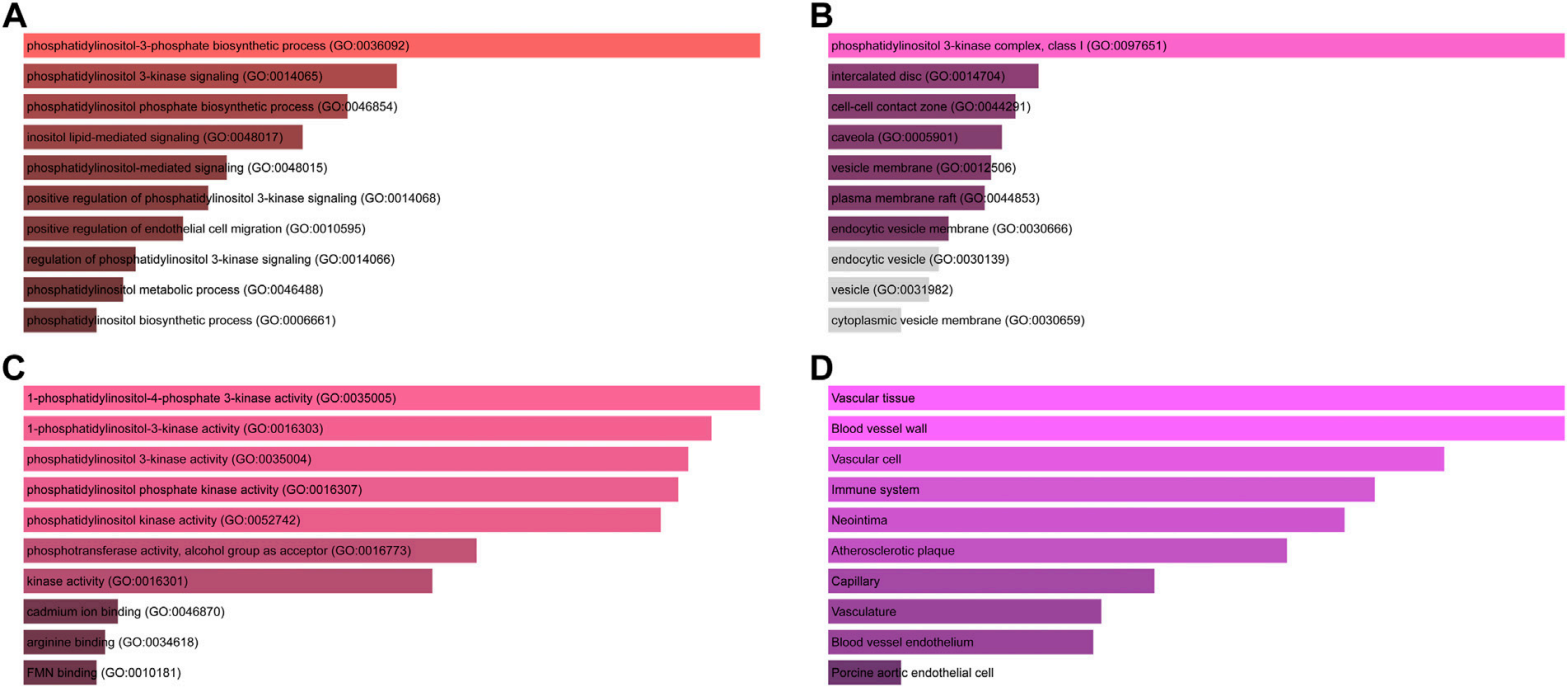
GO functional and tissue annotation for the common target genes of disease-compounds. **(A)** BP; **(B)** CC; **(C)** MF; **(D)** tissue. Ranking by-log10 (*p*-value).

Initial common target genes of disease-compound were discovered for GO enrichment analysis to categorise the potential functions of these important common target genes of disease-compounds. Four types of outcomes, namely, BP, CC, MF, and tissues, were created from the data. The top ten phrases for GO enrichment of common target genes of disease-compounds in each category are displayed. The common target genes of disease-compounds, primarily enriched in the BP category, were the phosphatidylinositol-3-phosphate biosynthetic process, phosphatidylinositol 3-kinase signalling, and the phosphatidylinositol phosphate biosynthetic process (Figure 4A and Supplementary Table S1). The CC category included the phosphatidylinositol 3-kinase complex, class I, intercalated disc, and the cell-cell contact zone (Figure 4B and Supplementary Table S2). MF category included 1-phosphatidylinositol-4-phosphate 3-kinase activity, 1-phosphatidylinositol-3-kinase activity, and phosphatidylinositol 3-kinase activity (Figure 4C and Supplementary Table S3). The common target genes of disease-compounds, primarily enriched in the Jensen tissue category, were vascular tissue, blood vessel wall, and vascular cell (Figure 4D and Supplementary Table S4).

#### 4.4.1 KEGG pathway analysis of common target genes of disease-compound

**FIGURE 5:**
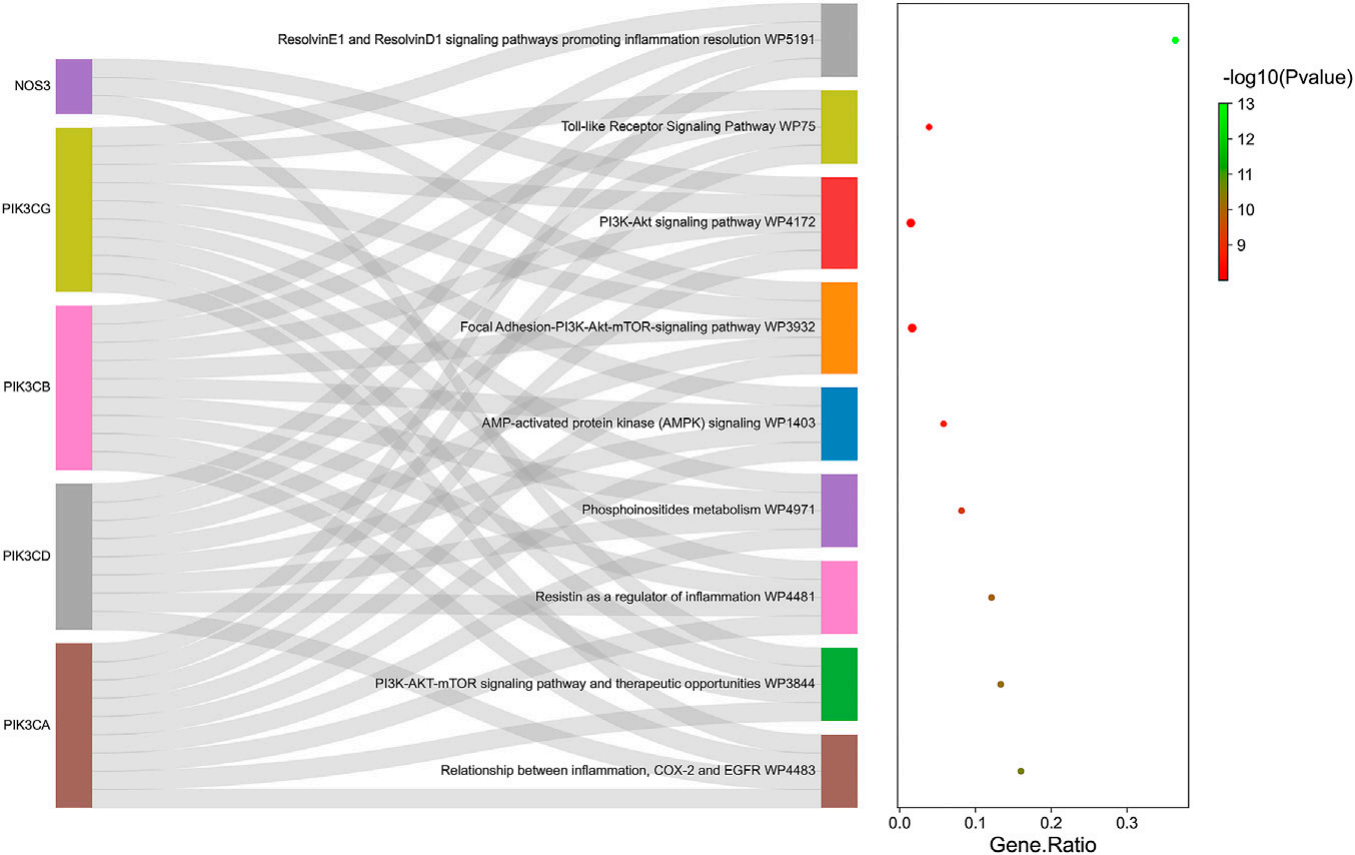
KEGG pathway enrichment analysis for the common target genes of disease-compound. Ranking by-log10 (*p*-value).

KEGG enrichment was then conducted on the common target genes of disease-compound. The common target genes of disease-compounds primarily enriched in the KEGG category were ResolvinE1 and ResolvinD1 signalling pathways (promoting inflammation resolution), the PI3K-Akt signalling pathway, the focal Adhesion-PI3K-Akt-mTOR-signalling pathway, AMP-activated protein kinase (AMPK) signalling, therapeutic opportunities, the PI3K-AKT-mTOR signalling pathway, as well as the relationship between inflammation, COX-2 and EGFR (Figure 5 and Table 1).

**TABLE 1:** Top ten KEGG pathways terms of common target genes of disease-compound.

Term	Overlap	*p*-value	Adjusted *p*-value	Odds Ratio	Combined Score	Genes
Relationship between inflammation, COX-2 and EGFR WP4483	4/25	2.84E-11	2.09E-09	1902.19	46,192.98	PIK3CA; PIK3CD; PIK3CB; PIK3CG
PI3K-AKT-mTOR signaling pathway and therapeutic opportunities WP3844	4/30	6.15E-11	2.09E-09	1536	36,113.53	PIK3CA; NOS3; PIK3CB; PIK3CG
Resistin as a regulator of inflammation WP4481	4/33	9.19E-11	2.34E-09	1376.897	31,821.11	PIK3CA; PIK3CD; PIK3CB; PIK3CG
Phosphoinositides metabolism WP4971	4/49	4.75E-10	5.38E-09	886.6222	19,033.66	PIK3CA; PIK3CD; PIK3CB; PIK3CG
AMP-activated protein kinase (AMPK) signaling WP1403	4/69	1.94E-09	1.97E-08	613.2	12,302.66	PIK3CA; PIK3CD; PIK3CB; PIK3CG
Focal Adhesion-PI3K-Akt-mTOR-signaling pathway WP3932	5/303	4.58E-09	3.59E-08	330.4698	6345.799	PIK3CA; NOS3; PIK3CD; PIK3CB; PIK3CG
PI3K-Akt signaling pathway WP4172	5/340	8.16E-09	5.55E-08	293.4179	5464.679	PIK3CA; NOS3; PIK3CD; PIK3CB; PIK3CG
Toll-like Receptor Signaling Pathway WP75	4/103	9.87E-09	6.29E-08	401.9192	7408.86	PIK3CA; PIK3CD; PIK3CB; PIK3CG
ResolvinE1 and ResolvinD1 signaling pathways promoting inflammation resolution WP5191	4/11	1.00E-13	2.51E-09	254.3333	9059.458	PIK3CA; PIK3CB; PIK3CD; PIK3CG

#### 4.4.2 Construction of the key CEP–gene–pathway-ARDS network

**FIGURE 6:**
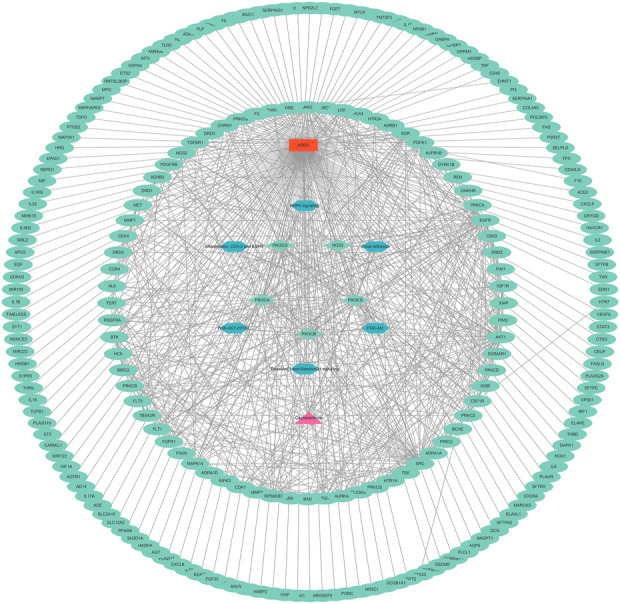
Key CEP–gene–pathway-ARDS network.

A key CEP–gene–pathway-ARDS network was constructed, including 152 nodes (5 targets and 6 pathways) and 744 links (Figure 6).

### 4.5 CEP alleviated oleic acid induced-ARDS lung injury in rats

**FIGURE 7:**
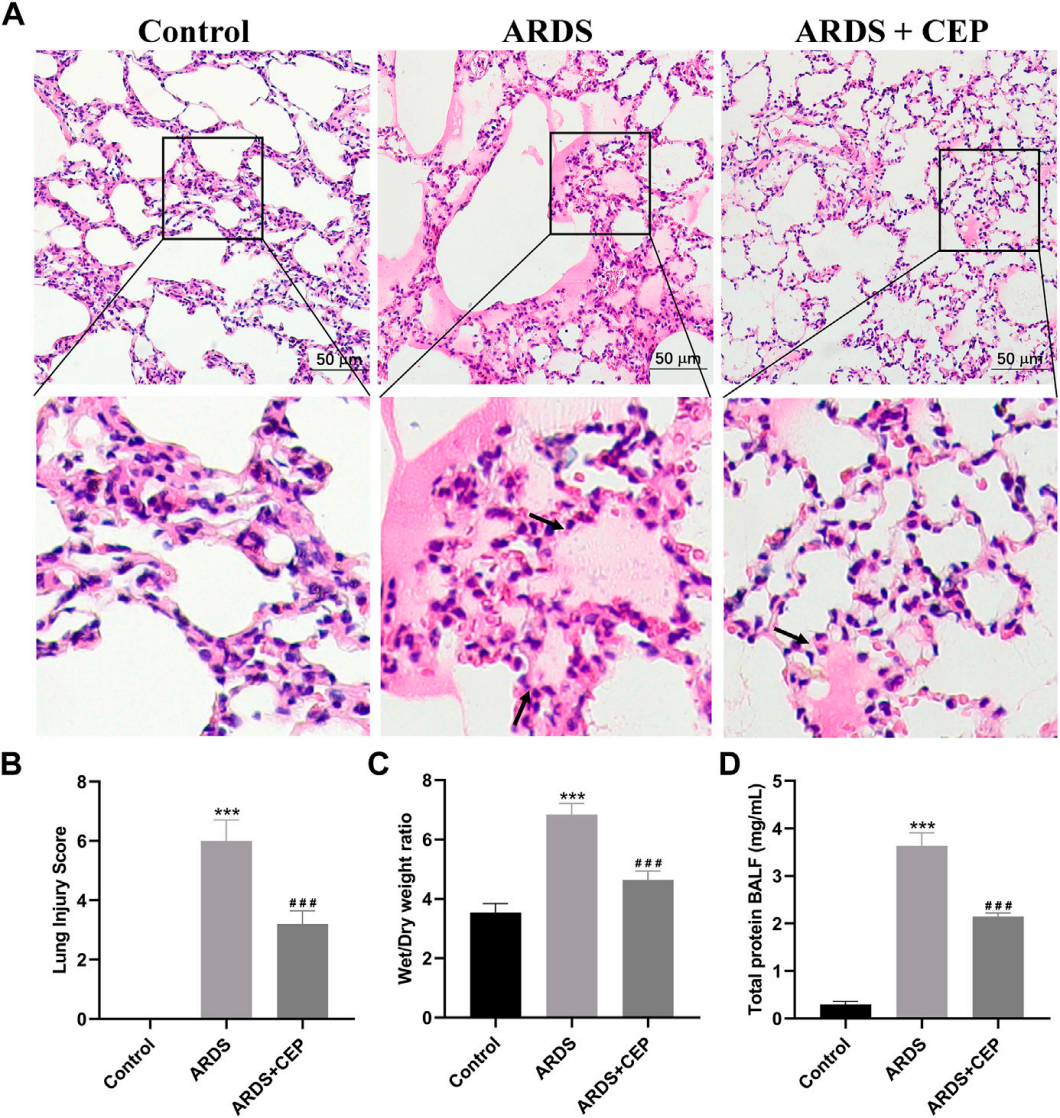
Evaluation of the CEP alleviation of oleic acid induced-ARDS lung injury *in vivo*. **(A)** Representative histological images of lung in each group (scale bar = 50 μm). Arrows represent exudative lesions and bleeding. **(B)** Lung injury scores according to the degree of lung damage in each group. **(C)** Wet/Dry weight ratios of lungs as an index of lung edema. **(D)** Total protein concentration in the BALF. Data are presented as the mean ± SD. ***p < 0.001, compared with the Control group; ^###^ *p* < 0.001, compared with the ARDS group.

H&E staining showed the pathological changes of lung tissue in each group. Lung histopathology in the ARDS group showed clear alveolar inflammatory cell infiltration, edema and interstitial thickening, which was significantly alleviated after CEP treatment (Figure 7A). The lung injury score quantification confirmed that oleic acid-induced severe lung damage and was substantially attenuated by treatment with CEP (Figure 7B). Lung edema was quantified using the Wet/Dry weight ratio (Figure 7C). As we expected, the Wet/Dry ratio of lung tissues in the ARDS group was higher than that in the Control group, and was significantly decreased after the CEP treatment. In addition, we detected the BALF protein concentration and found that the ARDS group BALF protein concentration significantly increased, while it reduced in the ARDS + CEP group (Figure 7D).

### 4.6 Effects of CEP on ARDS-induced inflammation

**FIGURE 8:**
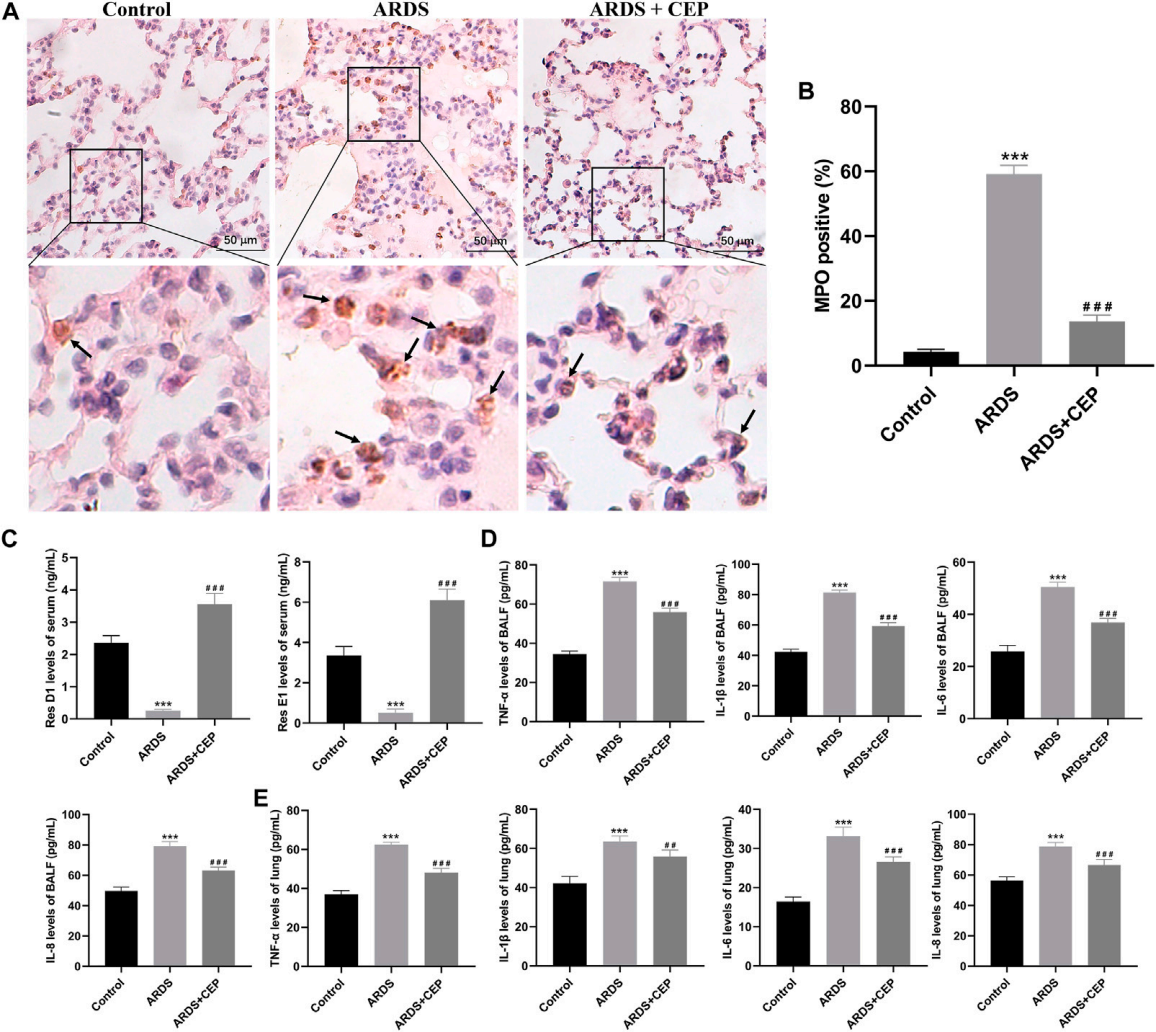
Effects of CEP on oleic acid induced-ARDS inflammation in rats. **(A,B)** Representative images of IHC staining for MPO (brown), a marker of neutrophils and their quantification in each group. Scale bar = 50 μm. **(C)** Levels of serum Res D1 and Res E1 assessed by ELISA. **(D,E)** Levels of TNF-α, IL-1β, IL-6, and IL-8 in BALF and lung tissues assessed by ELISA. Data are presented as the mean ± SD. ***p < 0.001, compared with the Control group; ^##^ *p* < 0.01, ^###^ *p* < 0.001, compared with the ARDS group.

To identify the inflammatory infiltrate, standard IHC was performed to stain for neutrophil-specific marker, MPO. MPO staining in lung tissues of each group was shown in Figure 8A. The positive expression of MPO in the ARDS + CEP group was significantly lower than that in the ARDS group (Figure 8B). However, the levels of serum Res D1 and Res E1 were significantly higher in the Control and ARDS + CEP group, compared with the ARDS group (Figure 8C). In terms of proinflammatory cytokines, the levels of TNF-α, IL-1β, IL-6, and IL-8 in BALF and lung tissues were higher in the ARDS group compared with the Control group; moreover, CEP treatment decreased their levels (Figures 8D,E).

## 5 Discussion

ARDS is a common critical illness, characterised by a large number of inflammatory reactions, which play an important role in its occurrence and development (Dickson et al., 2016). Therefore, effective suppression of inflammatory response is the basic treatment strategy for ARDS. TCM is widely used in the treatment of various diseases, among which it shows unique advantages in the treatment of lung diseases (Jin et al., 2021). However, several issues remain to be resolved, among them, the effective components and target genes of TCM have always been the key issues in the research of TCM (Jin et al., 2021). Based on NA and *in vivo* experimental verification, this study confirmed that CEP, an active component of TCM, played a significant role in improving the treatment of inflammation in ARDS.

The success of a medicinal drug depends not only on good efficacy, but also on the acceptable characteristics of ADME (Vaou et al., 2021). The ADME characteristics of CEP were analyzed based on the SwissADME database, as well as parameters including good lipophilicity, water solubility, GI absorption and bioavailability, which provide a basis for CEP as a molecular therapy for disease. We carried out NA analysis in order to further evaluate the therapeutic effect of CEP on ARDS and predict its therapeutic target. Firstly, we constructed the PPI network that identified PIK3CA PIK3CB, PIK3CD, PIK3CG, NOS3, and REN are core targets in the context of CEP-mediated ARDS treatment. Subsequently, in order to clarify the multiple mechanisms of CEP on ARDS, we analyzed the enrichment of these targets by GO and KEGG. GO analysis showed that the treatment of CEP on ARDS was mainly related to phosphatidylinositol-3-phosphate (PI3P), phosphatidylinositol 3-kinase (PI3K) and the phosphatidylinositol phosphate (PIP) biosynthetic process. For PI3P, many steps of autophagy require the involvement of PI3P (Steinfeld et al., 2021). Many studies have shown that autophagy is associated with various lung diseases, including ARDS (Schuliga et al., 2021; Tomer et al., 2021). For PI3K, it plays an important role in inflammatory response, which promotes the production of a variety of pro-inflammatory cytokines (Cianciulli et al., 2016). In addition, PIP was reported to be essential in vesicular trafficking, organelle biogenesis and autophagy (Nascimbeni et al., 2017). Further KEGG analyses found that the role of CEP was mainly concentrated in the inflammatory factor signalling pathway of ResE1 and ResD1, as well as the apoptotic signalling pathway of PI3K-Akt. Next, in the rat ARDS model induced by oleic acid, it was further confirmed that CEP alleviated lung injury by inhibiting lung inflammatory response.

The animal model induced by oleic acid is a well-recognized animal model of ARDS (Fan et al., 2015; Huang et al., 2022a; Matute-Bello et al., 2008; Puuvuori et al., 2022). The destruction of alveolar capillary barrier, microvascular thrombosis and massive inflammation, are the main pathophysiological features of oleic acid-induced ARDS (Matute-Bello et al., 2008). Our study demonstrated that CEP treatment significantly ameliorated lung inflammation, reduced pulmonary edema, and improved histologic lung injury scores. MPO is a pro-inflammatory enzyme, mainly produced by activated neutrophils, which can promote the aggravation and prolongation of inflammation (Koyani et al., 2015). Previous studies have found that oleic acid, injected into lung tissue, can stimulate neutrophil accumulation (Ito et al., 2005; El Ayed et al., 2017). We found that CEP can significantly reduce the expression of MPO positive cells in lung tissue. In the above KEGG enrichment, significant enrichment belongs to ResE1 and ResD1 signalling pathways promoting inflammation resolution. Resolvins are potent short-lived autacoids that belong to a novel family of bioactive lipids and participate in the resolution process (Xie et al., 2016). Resolvins play an important role in orchestrating inflammation resolution (Guo et al., 2020). ResD1 and ResE1, as important members of the Resolvins family, have received more and more attention in the field of lung injury research (Seki et al., 2010; Wang et al., 2011; Thankam et al., 2022). Previous studies have shown that ResD1 protects the integrity of endothelial cell adhesion and barrier function from inflammatory mediators by inhibiting ROS production and preventing SHP2 inactivation (Wang et al., 2011). ResE1 is produced at the site of inflammation through transcellular metabolism and has been shown to effectively inhibit the migration of neutrophils across endothelial cells (Siddiquee et al., 2019). Our study found that the serum levels of ResD1 and ResE1 in CEP-treated rats were significantly increased. The proinflammatory mediator promote recruitment of inflammatory cells particularly, neutrophils and eosinophils, into the airways by its direct and indirect chemotactic properties (Arora et al., 2022a). IL-1β is a pro-inflammatory cytokine, which can trigger immune and inflammatory responses, and can be synthesised and secreted by a variety of cells (He et al., 2015). Pro-inflammatory cytokines IL-6 and IL-8 are important mediators of inflammation locally and systemically (Wang et al., 2009). Antioxidants can prevent the enzymatic and non-enzymatic production of oxidant molecules and protect against the injurious effects of ROS (Arora et al., 2022b). As CEP can reduce the production of oxidants, it is reasonable to speculate that CEP may very likely act through oxidant production inhibition to exert its effects on downregulating inflammatory molecule expression (Chang et al., 2016). The expression levels of TNF-α, IL-1β, IL-6, and IL-8 in lung tissue and BALF were detected by ELISA *in vivo*. The results showed that CEP inhibited the expression of these inflammatory factors. Therefore, the data indicates that CEP effectively suppresses the inflammatory response of lung injury and confirms the results relating to the function of enrichment analysis.

Although this study has obtained a lot of effective information on the basis of NA and has been verified by experiments, there are still some limitations. Firstly, we collected all the relevant targets of CEP and ARDS from databases (as much as possible), however, we were not able to collect the latest research results. Secondly, although CEP can be administered safely over a range of doses without evident side effects, our study used only a single dose (10 mg/kg) for the CEP treatment. Thirdly, in terms of experimental verification, we only confirmed the therapeutic effect of CEP on the inflammatory response of ARDS, and no further research on the mechanism has been carried out, which will be the focus of future research work.

## 6 Conclusion

To sum up, this study preliminarily determined the key targets and important signalling pathways of CEP in the treatment of ARDS, based on NA. We verified the accuracy of the above results through *in vivo* experiments, which laid a theoretical foundation for the clinical application of CEP.

## Data Availability

The datasets presented in this study can be found in online repositories. The names of the repository/repositories and accession number(s) can be found in the article/Supplementary Material.

## Abbreviations

ADME, absorption, distribution, metabolism, and excretion; ARDS, acute respiratory distress syndrome; BALF, broncho-alveolar lavage fluid; BP, biological processes; CC, cellular components; CEP, cepharanthine; DisGeNET, disease target genes; ELISA, enzyme-linked immunosorbent assay; GO, gene ontology; H&E, hematoxylin-eosin; IHC, immunohistochemical; KEGG, kyoto encyclopedia of genes and genomes; MF, molecular function; MPO, myeloperoxidase; NA, network Analysis; PI3P, phosphatidylinositol-3-phosphate; PI3K, phosphatidylinositol 3-kinase; PIP, phosphatidylinositol phosphate; PPI, protein-protein interactions; Res D1, ResolvinD1; Res E1, ResolvinE1; SARS-CoV-2, severe acute respiratory syndrome coronavirus 2; TCM, traditional Chinese medicines.
