# Supplementary material for: Network analysis-based strategy to investigate the protective effect of cepharanthine on rat acute respiratory distress syndrome
Source: Front Pharmacol. 2022 Oct 26;13:1054339. doi: 10.3389/fphar.2022.1054339 (PMC9645439; doi:10.3389/fphar.2022.1054339)
Supplement: Supplementary file 1 [file Table1.docx]

Table S1. Top ten biological processes terms of common target genes of disease-compound

| Term | Overlap | P-value | Adjusted P-value | Odds Ratio | Combined Score | Genes |
| --- | --- | --- | --- | --- | --- | --- |
| phosphatidylinositol-3-phosphate biosynthetic process (GO:0036092) | 4/8 | 1.57E-13 | 3.61E-11 | 9995 | 294650.5 | PIK3CA; PIK3CD; PIK3CB; PIK3CG |
| phosphatidylinositol 3-kinase signaling (GO:0014065) | 4/34 | 1.04E-10 | 1.19E-08 | 1330.933 | 30592.38 | PIK3CA; PIK3CD; PIK3CB; PIK3CG |
| phosphatidylinositol phosphate biosynthetic process (GO:0046854) | 4/42 | 2.51E-10 | 1.92E-08 | 1050.316 | 23217.43 | PIK3CA; PIK3CD; PIK3CB; PIK3CG |
| inositol lipid-mediated signaling (GO:0048017) | 4/51 | 5.60E-10 | 3.21E-08 | 848.8085 | 18081.92 | PIK3CA; PIK3CD; PIK3CB; PIK3CG |
| phosphatidylinositol-mediated signaling (GO:0048015) | 4/71 | 2.17E-09 | 9.96E-08 | 594.8358 | 11864.81 | PIK3CA; PIK3CD; PIK3CB; PIK3CG |
| positive regulation of phosphatidylinositol 3-kinase signaling (GO:0014068) | 4/77 | 3.03E-09 | 1.16E-07 | 545.7808 | 10705.79 | PIK3CA; PIK3CD; PIK3CB; PIK3CG |
| positive regulation of endothelial cell migration (GO:0010595) | 4/86 | 4.75E-09 | 1.55E-07 | 485.6585 | 9307.982 | NOS3; PIK3CD; PIK3CB; PIK3CG |
| regulation of phosphatidylinositol 3-kinase signaling (GO:0014066) | 4/106 | 1.11E-08 | 3.17E-07 | 390.0392 | 7144.512 | PIK3CA; PIK3CD; PIK3CB; PIK3CG |
| phosphatidylinositol metabolic process (GO:0046488) | 4/112 | 1.39E-08 | 3.53E-07 | 368.2593 | 6663.489 | PIK3CA; PIK3CD; PIK3CB; PIK3CG |
| phosphatidylinositol biosynthetic process (GO:0006661) | 4/126 | 2.23E-08 | 5.11E-07 | 325.7705 | 5739.578 | PIK3CA; PIK3CD; PIK3CB; PIK3CG |
