# Supplementary material for: Network analysis-based strategy to investigate the protective effect of cepharanthine on rat acute respiratory distress syndrome
Source: Front Pharmacol. 2022 Oct 26;13:1054339. doi: 10.3389/fphar.2022.1054339 (PMC9645439; doi:10.3389/fphar.2022.1054339)
Supplement: Supplementary file 2 [file Table2.docx]

Table S2. Top ten cellular components terms of common target genes of disease-compound

| Term | Overlap | P-value | Adjusted P-value | Odds Ratio | Combined Score | Genes |
| --- | --- | --- | --- | --- | --- | --- |
| phosphatidylinositol 3-kinase complex, class I (GO:0097651) | 2/5 | 7.50E-07 | 1.27E-05 | 3331.833 | 46990.97 | PIK3CA; PIK3CG |
| intercalated disc (GO:0014704) | 1/31 | 0.009265 | 0.068998 | 133.0933 | 623.0766 | PIK3CA |
| cell-cell contact zone (GO:0044291) | 1/47 | 0.014019 | 0.068998 | 86.73043 | 370.1082 | PIK3CA |
| caveola (GO:0005901) | 1/60 | 0.017868 | 0.068998 | 67.57627 | 271.9786 | NOS3 |
| vesicle membrane (GO:0012506) | 1/73 | 0.021704 | 0.068998 | 55.33889 | 211.9631 | NOS3 |
| plasma membrane raft (GO:0044853) | 1/82 | 0.024352 | 0.068998 | 49.1679 | 182.6656 | NOS3 |
| endocytic vesicle membrane (GO:0030666) | 1/158 | 0.046479 | 0.112878 | 25.27006 | 77.54756 | NOS3 |
| endocytic vesicle (GO:0030139) | 1/189 | 0.055384 | 0.11769 | 21.07021 | 60.96601 | NOS3 |
| vesicle (GO:0031982) | 1/226 | 0.065921 | 0.124517 | 17.57244 | 47.78472 | NOS3 |
| cytoplasmic vesicle membrane (GO:0030659) | 1/380 | 0.108732863 | 0.184845867 | 10.35092348 | 22.96726253 | NOS3 |
