# Supplementary material for: Network analysis-based strategy to investigate the protective effect of cepharanthine on rat acute respiratory distress syndrome
Source: Front Pharmacol. 2022 Oct 26;13:1054339. doi: 10.3389/fphar.2022.1054339 (PMC9645439; doi:10.3389/fphar.2022.1054339)
Supplement: Supplementary file 3 [file Table3.docx]

Table S3. Top ten molecular function terms of common target genes of disease-compound

| Term | Overlap | P-value | Adjusted P-value | Odds Ratio | Combined Score | Genes |
| --- | --- | --- | --- | --- | --- | --- |
| 1-phosphatidylinositol-4-phosphate 3-kinase activity (GO:0035005) | 4/7 | 7.87E-14 | 1.81E-12 | 13327.33 | 402123.9 | PIK3CA; PIK3CD; PIK3CB; PIK3CG |
| 1-phosphatidylinositol-3-kinase activity (GO:0016303) | 4/10 | 4.72E-13 | 5.43E-12 | 6662.667 | 189095.2 | PIK3CA; PIK3CD; PIK3CB; PIK3CG |
| phosphatidylinositol 3-kinase activity (GO:0035004) | 4/12 | 1.11E-12 | 8.53E-12 | 4996.5 | 137523.6 | PIK3CA; PIK3CD; PIK3CB; PIK3CG |
| phosphatidylinositol phosphate kinase activity (GO:0016307) | 4/13 | 1.61E-12 | 9.24E-12 | 4441.111 | 120604.3 | PIK3CA; PIK3CD; PIK3CB; PIK3CG |
| phosphatidylinositol kinase activity (GO:0052742) | 4/15 | 3.07E-12 | 1.41E-11 | 3633.273 | 96317.52 | PIK3CA; PIK3CD; PIK3CB; PIK3CG |
| phosphotransferase activity, alcohol group as acceptor (GO:0016773) | 4/75 | 2.72E-09 | 1.04E-08 | 561.2113 | 11068.66 | PIK3CA; PIK3CD; PIK3CB; PIK3CG |
| kinase activity (GO:0016301) | 4/112 | 1.39E-08 | 4.55E-08 | 368.2593 | 6663.489 | PIK3CA; PIK3CD; PIK3CB; PIK3CG |
| cadmium ion binding (GO:0046870) | 1/5 | 0.001499 | 0.00431 | 999.5 | 6499.559 | NOS3 |
| arginine binding (GO:0034618) | 1/8 | 0.002398 | 0.006128 | 571.0571 | 3445.29 | NOS3 |
| FMN binding (GO:0010181) | 1/11 | 0.003296 | 0.007517 | 399.68 | 2284.211 | NOS3 |
