# Supplementary material for: Network analysis-based strategy to investigate the protective effect of cepharanthine on rat acute respiratory distress syndrome
Source: Front Pharmacol. 2022 Oct 26;13:1054339. doi: 10.3389/fphar.2022.1054339 (PMC9645439; doi:10.3389/fphar.2022.1054339)
Supplement: Supplementary file 4 [file Table4.docx]

Table S4. Top ten tissue terms of common target genes of disease-compound

| Term | Overlap | P-value | Adjusted P-value | Odds Ratio | Combined Score | Genes |
| --- | --- | --- | --- | --- | --- | --- |
| Vascular tissue | 2/30 | 3.25E-05 | 0.002064 | 356.5357 | 3684.5 | NOS3; REN |
| Blood vessel wall | 2/30 | 3.25E-05 | 0.002064 | 356.5357 | 3684.5 | NOS3; REN |
| Vascular cell | 2/43 | 6.74E-05 | 0.002851 | 243.3293 | 2337.304 | NOS3; REN |
| Immune system | 4/1046 | 1.03E-04 | 0.003126 | 36.3762 | 334.1356 | NOS3; PIK3CD; REN; PIK3CG |
| Neointima | 2/58 | 1.23E-04 | 0.003126 | 178.0179 | 1602.675 | NOS3; REN |
| Atherosclerotic plaque | 2/69 | 1.74E-04 | 0.003691 | 148.709 | 1286.963 | NOS3; REN |
| Capillary | 2/103 | 3.89E-04 | 0.007052 | 98.4802 | 773.3341 | NOS3; REN |
| Vasculature | 2/121 | 5.36E-04 | 0.007941 | 83.5084 | 628.9443 | NOS3; REN |
| Blood vessel endothelium | 2/124 | 5.63E-04 | 0.007941 | 81.44262 | 609.4128 | NOS3; REN |
| Porcine aortic endothelial cell | 1/6 | 0.001799 | 0.020768 | 799.56 | 5053.709 | NOS3 |
